# Are Danish vocational schools ready to implement “smoke-free school hours”? A qualitative study informed by the theory of organizational readiness for change

**DOI:** 10.1186/s43058-021-00140-x

**Published:** 2021-04-09

**Authors:** Anneke Vang Hjort, Michael Schreuders, Kathrine Højlund Rasmussen, Charlotte Demant Klinker

**Affiliations:** 1 Health Promotion Research, Steno Diabetes Center Copenhagen, Niels Steensens Vej 2, 2820 Gentofte, Denmark; 2 National Institute of Public Health, University of Southern Denmark, Studiestræde 6, 1455 Copenhagen, Denmark; 3 Department of Public Administration & Sociology, Erasmus School of Behavioral and Social Studies, Erasmus University Rotterdam, 3000 Rotterdam, DR The Netherlands; 4 Prevention, The Danish Heart Foundation, Vognmagergade 7, 1120 Copenhagen, Denmark

**Keywords:** Vocational education and training, School tobacco policy, Smoke-free school hours, Organizational readiness for change, Facilitators and barriers, Policy implementation

## Abstract

**Background:**

The smoking prevalence is high among students enrolled in vocational education and training, which is considered a lower level of education. The school tobacco policy regarding smoke-free school hours stipulates that students and staff are not allowed to smoke during school hours—inside or outside school premises—and it might be an effective intervention for reducing smoking in vocational schools. For school tobacco policies to be effective, they must be appropriately implemented. A primary predictor for successful implementation is organizational readiness for change. This study seeks to identify and understand the barriers to and facilitators for developing organizational readiness to implement smoke-free school hours in Danish vocational schools.

**Methods:**

Semi-structured interviews and focus groups were carried out with managers and teachers (*n* = 22 participants) from six vocational schools. The interview guides were informed by “A theory of organizational readiness for change” developed by Weiner, which was also used as a framework to analyze the data.

**Results:**

We identified 13 facilitators and barriers. Nine factors acted as facilitators, including the following: believing that health promotion is a school role and duty; believing that society and workplaces are becoming more smoke-free, and believing that smoke-free school hours is a beneficial strategy to achieve fewer educational interruptions. Additional facilitators include establishing clear rules for sanctioning and enforcement, developing a joint understanding about smoke-free school hours, developing skills to deal with student responses to smoke-free school hours, establishing social alternatives to smoking, offering smoking cessation help, and mandating smoke-free school hours by law. Four organizational norms, practices, or discourses acted as barriers: believing that smoke-free school hours violate personal freedom, believing that students have more important problems than smoking, believing that it is difficult to administer the level of enforcement, and believing that the enforcement of smoke-free school hours negatively influences student-teacher relations.

**Discussion:**

Our results suggest that developing organizational readiness before adopting a comprehensive tobacco policy such as smoke-free school hours is important for successful implementation. Further research should investigate how to strengthen the facilitators for and counter the barriers to developing readiness for implementing smoke-free school hours.

**Supplementary Information:**

The online version contains supplementary material available at 10.1186/s43058-021-00140-x.

Contributions to the literature

- Our study contributes with a theory-based approach to the study of change readiness in relation to implementing smoke-free school policies and further identifies specific actions and concerns that need to be addressed to develop change readiness.
- We investigated implementation challenges and potentials within the setting of vocational schools, which is an understudied setting.
- Findings from this study have been used to inform the development of an intervention supporting the implementation of smoke-free school hours in Danish vocational schools. This study thus provides an example of how new knowledge can bridge the gap between research and practice.

## Background

As in too many European countries, the smoking prevalence among Danish young adults is especially high among those with lower levels of education [1]: The proportion of daily smokers in vocational education and training (VET) is 29% [2] compared to 9% among peers in the academically oriented general upper-secondary education [3]. Vocational schools provide a practical upper-secondary education for a specific service or industry (e.g., carpenter, hairdresser, health care assistant, chef), which is characterized by a combination of traditional in-school education and out-of-school apprenticeship at a workplace. Most students attending Danish vocational schools are young adults between the ages of 15–24 years (64%), but school enrollments reflect substantial diversity in age, as some students enroll much later in their adult lives [4].

No legislative school tobacco policy (STP) applied to Danish vocational schools at the time of this study. However, in December 2019, the Danish parliament decided to ratify smoke-free school hours (SFSH) beginning in August 2021 for all educational institutions with students under the age of 18, also including vocational schools. The basic rationale for STPs and SFSH is the same: restricting smoking behavior as a means to prevent smoking initiation, smoking continuation, and exposure to second-hand smoke among adolescents and young adults [5, 6]. The difference between the two is that SFSH entail a complete smoking ban for all students and staff during the entire school day, whereas STPs do not prohibit smoking outside the school area. The possibility of smoking outside the school area is a major challenge for STPs because students may relocate their smoking to just outside the school entrance, increasing smoking visibility [7, 8]. SFSH is intended to aid in overcoming this challenge by prohibiting smoking during school hours altogether. As such, SFSH is more comprehensive and restrictive than usual STPs.

The effectiveness of SFSH depends on how well the policy is implemented [9, 10]. As many implementation definitions exist, our conceptualization is important. In this study, implementation is defined as the social organization of the work of bringing an intervention into routine practice, which involves dynamic and contingent interactions within a context over time [11, 12]. In the case of SFSH, implementation is a school organizational process in which the end goal is to incorporate the policy into practice equivalent to other implemented school policies (e.g., students must attend to class on time). Hence, staff and managers must enact and enforce the policy as part of their professional tasks, and students experience the policy as an accepted part of their everyday school life. Enforcement is thus a significant part of policy implementation [9, 10, 13–15].

Another important aspect to implementation is change readiness. Some scholars suggest that organizational readiness is a primary predictor for successful implementation [16], whereas others have shown that failure to establish sufficient readiness accounts for one half of all unsuccessful organizational change efforts [17]. A recent review by Weiner et al. [18] presents the state of the art of theoretical definitions of change readiness and evidence on how and why it matters. The authors conclude that change readiness is often operationalized as individual attitudes toward change (how people think or feel about change), which does not correspond to the everyday discourse on readiness, which connotes a state of preparedness to do something or for something (how prepared people are to act or respond to support change implementation). Despite theoretical ambiguities, the authors highlight conditions, which promote organizational readiness, e.g., trust in leadership, perceived organizational support, and flexible organizational policies and procedures. Also, timely and accurate change communication and employee involvement in the change process might be positively associated with readiness. Finally, individual characteristics such as psychological ownership and job satisfaction might promote change readiness [18]. Likewise, the literature on implementing health promotive initiatives in schools has highlighted that preparation for change within the school organization is crucial for successful implementation [19–21]. Developing organizational readiness involves a whole-school approach, in which leadership is especially important to stimulating motivation for change and for providing support for teachers in their role as change agents [20]. Thus, building readiness for implementing SFSH is important so that school staff and managers have sufficient skills and commitment to take on the implementation work.

In many secondary schools, STPs are not well implemented [22–25]. A systematic review found that key contextual factors for successfully implementing tobacco- and substance-use interventions in secondary schools are a positive organizational climate, adequate training, and teachers’ and students’ motivation [26]. Barriers to implementation include heavy workloads, budget cuts and lack of resources or support [26]. Additionally, a realist review contended that widespread inconsistent enforcement is the reason for implementation failure and showed that staff enforcement depends on whether they (1) believe that STP enforcement is their role and duty, (2) have confidence to deal with students’ negative responses when enforcing the rules, and (3) experience that enforcement has a positive impact on students [9]. A recent qualitative study [27] found that the insufficient readiness to implement SFSH in Dutch secondary schools can be explained by hindering beliefs (e.g., that smoking is not perceived as a pressing issue, that SFSH interfere with adolescents’ autonomy and would be impossible to enforce consistently). Because most of the existing literature has focused on secondary schools (adolescents) or tertiary schools (young adults with higher education), it is currently unclear if comparable facilitators and barriers to implementation, enforcement, and change readiness are present at vocational schools with a lower educated population group. For example, how does the close collaboration between vocational schools and apprenticeship workplaces affect school motivation to implement SFSH and which factors are perceived as important when enforcing the rules among vocational school students? A smoke-free society can be achieved only if we manage to prevent people with lower levels of education from smoking initiation and continuation. Therefore, it is important to know more about what facilitates or hinders the implementation of SFSH in the vocational school context.

### Theoretical framework

Implementation scholars emphasize the need to base implementation studies on theory [28]. We used the social-cognitive “A theory of organizational readiness for change” [16] as the guiding theory to investigate readiness for implementing SFSH in Danish vocational schools. Organizational readiness for change is defined as a shared psychological state within an organization: If there is sufficient organizational readiness for change, it means better change-related efforts and better chances of successful implementation [16]. Organizational readiness for change consists of change commitment and change efficacy. Change commitment is operationalized as staff and managers’ willingness to and motivation for pursuing the courses of action involved in change implementation, as well as the perceived benefits of doing so [16]. Change efficacy is defined as beliefs in skills, resources, and abilities to work together to execute the actions of change implementation [16]. As such, the theory corresponds to the everyday discourse of readiness that is a state of preparedness for future action [18].

Different contexts create different influences on the way in which school agents can function and on the complexity of introducing change [29]. Organizational readiness for change is more likely accomplished if the intervention aligns with school values, policies, and practices [21]. In the theory of organizational readiness for change, context is mediated through the psychological concepts of change commitment and change efficacy [16]: People’s judgments about their willingness or ability to implement a policy are not formed in a social vacuum but shaped by, for example, discourses and practices within a school organization. As such, change commitment and change efficacy reflect both personal beliefs and context (i.e., organizational norms, practices, and values). The organizational readiness for change theory thus provides a theoretical lens whereby we can study how different beliefs and contexts hinder or enable preparedness to practice SFSH. To our knowledge, this is the first study to use organizational readiness for change theory to investigate readiness to implement STPs in general and specifically related to SFSH in vocational schools.

### Study aim

The aim of this study was to identify and investigate facilitators for and barriers to developing organizational readiness to implement SFSH at vocational schools. The knowledge generated from this study has been used to inform the development of an intervention about how to support Danish vocational schools in implementing SFSH.

## Methods

Both Standards for Reporting Qualitative Research (SRQR) and Consolidated Criteria for Reporting Qualitative Research (COREQ) have been used as checklists in reporting of this qualitative study — see Additional file 1.

### Study design, participants, and recruitment

Danish VET includes more than 100 different educational programs and is divided into four main subject areas: (1) technology, construction, and transportation; (2) administration, commerce, and business service; (3) food, agriculture, and hospitality; and (4) care, health, and pedagogy. We used a purposive sampling strategy [30] to recruit a heterogeneous sample of schools representing all four vocational main subject areas and different geographical locations (three out of five Danish regions). Further, our preselected criteria were to include three schools with and three schools without experiences implementing SFSH, as they were hypothesized to represent different perspectives with regard to change readiness. Because at the time of this study only two vocational schools had experiences with implementing SFSH, it was decided to include a “production school” with experiences implementing SFSH in the sample of schools. Production schools target young adults under the age of 25 and serve a preparatory purpose to motivate “non-academic” students toward upper-secondary education, mainly VET, by providing a practical learning environment [31]. Production schools are thus comparable to vocational schools.

A participation request was sent to all six schools by email and followed up by telephone to explain the purpose, methods and scope of the study. All invited schools (*n* = 6) agreed to participate. Managers and teachers were included in the study, as they have different interfaces, challenges, and opportunities when implementing SFSH. Managers were recruited to participate in semi-structured interviews, and teachers participated in focus groups. Semi-structured interviews are useful for gaining an in-depth understanding of participants’ experiences and reasons for acting [32] and were therefore used to provide knowledge related to the decision-making and organizational procedures of management (e.g., communicative strategies) in relation to implementing/potentially implementing SFSH. Focus groups are a useful method for dialog, discussion or consensus among participants, which can reflect organizational discourses [33] and were chosen as a method to gain insights into teachers’ practices, as they are expected to play a larger role in the daily enforcement of SFSH. At two schools, two managers asked to participate in the management interview, and as a result, those interviewers were conducted as group interviews while still using the management interview guide. One focus group with teachers was carried out with only one teacher because no other teachers were able to participate and therefore had the format of a semi-structured interview; however, it followed the interview guide used for focus groups with minor amendments. Altogether 6 interviews and 6 focus groups were conducted with a total of 22 participants, including 8 managers and 14 teachers. An overview of the school and participant characteristics are shown in Table 1.

**Table 1:** Overview of study participants and school characteristics (based on 2018 data)

	School A	School B	School C	School D	School E	School F
Main subject area	Production school	Care, health, and pedagogy	Care, health, and pedagogy	Technology, construction, and transportation	Food, agriculture, and hospitality	Administration, commerce, and business service
Region	Southern Denmark	Southern Denmark	Capital	Zealand	Zealand	Southern Denmark
School tobacco policy and/or experience with smoke-free school hours	Smoke-free school hours since 2013	Established smoke-free school hours in 2016 and withdrew the policy after 6 months. Smoking allowed at designated areas on school grounds (outdoors)	Smoke-free school hours since 2013	Smoking allowed on school grounds (outdoors)	Smoking only allowed outside school area	Smoking allowed at designated areas on school grounds (outdoors)
Participants (*n*) and their school positions	Principal manager	Principal manager and school health responsible	Principal manager	Departmental manager	Principal manager and departmental manager	Principal manager
	2 Teachers	1 Teacher	2 Teachers	3 Teachers	4 Teachers	2 Teachers

### Data collection and researcher characteristics

The semi-structured interviews and focus group interviews were based on interview guides informed by the theory of organizational readiness for change [16], as well as knowledge related to implementation capacity at Danish vocational schools obtained from previous work [34–36] and more general implementation research on the contextual factors associated with successful implementation [26, 37, 38]. The general ORIC-scale [39] covers many of the same themes as our interview guides, but we found it necessary to develop a context-specific assessment for our qualitative research aim. Additional file 2 contains the interview guides, thus our operationalization of theory to practice. Both the focus groups and semi-structured interviews included questions about the participants’ professional work tasks and their experiences integrating STPs into school practice. Further, all participants were asked specifically about their motivation, willingness, and commitment to implement SFSH as well as their concerns, barriers, and challenges. The interviews were carried out in Danish and the interview guides (Additional file 2) has been translated to English for the purpose of this paper. The translation of English concepts to Danish interview questions is not considered to alter nor deviate from the original meaning behind the theory of organizational readiness.

The interviews were conducted from November 2017 to January 2018 in locations chosen by the participants based on where they felt comfortable (e.g., an office with a closed door), and the interviews lasted 65 min on average (range, 48–114 min). Eleven interviews were carried out by the same interviewer (AVH, MSc, female), while one semi-structured interview with the manager from school C was led by another interviewer (MSc, male), but AVH acted as second interviewer. Both interviewers have education in and experience with qualitative methods and were employed as research assistants at Steno Diabetes Center Copenhagen and The University of Southern Denmark, respectively. Neither the interviewers nor the research team knew the study participants prior to the study commencement other than from telephone/email correspondence during recruitment.

Prior to each interview, the participants received written and verbal explanations of the study, including the study purpose, a statement that participation was voluntary and information about data confidentiality. Oral informed consent was obtained from all participants, which is considered appropriate according to Danish legislation [40]. All interviews including the informed consent were audio recorded and transcribed verbatim. Personal data (e.g., names) were pseudo-anonymized, and transcripts were stored in a secured digital folder accessible only by the research team. The study was reported to Capital Region of Denmark’ legal center for personal data handling according to the Danish data protection legislation and GDPR, journal number 2012-58-0004.

### Coding and data analysis

The organizational readiness for change theory was used as a framework to analyze the participants’ perceptions of the facilitators for and barriers to developing either change commitment or change efficacy. As a first step, AVH read all transcripts in-depth to obtain an understanding of the data as a whole. AVH then coded the transcripts using structuring content analysis in NVIVO 11. The objective of this method is to segregate transcripts into distinct manageable units (“meaning units”) [41]. A deductive approach was used in the coding and analysis process in which all (relevant) data were categorized as either (1) a facilitator or a barrier to (2) change commitment or change efficacy — see Table 2.

**Table 2:** Coding and analysis strategy matrix — barriers to and facilitators of change commitment and change efficacy

	Change commitment	Change efficacy
**Facilitator**	Meaning units about what is meaningful or beneficial about implementing smoke-free school hours.	Meaning units about what makes it possible or easier to practice smoke-free school hours.
**Barrier**	Meaning units about what is meaningless or negative about implementing smoke-free school hours.	Meaning units about what makes it impossible or hinders the practice of smoke-free school hours.

Implementation literature recognizes that the same factor can sometimes be considered both a barrier and a facilitator [42] — for example, in one organization, staff members have enough competences and confidence to execute the actions involved in change implementation (facilitator), whereas in another organization, they do not have enough competences and confidence (barrier) [43]. In this study, we classified factors as either facilitators or barriers based on the respondent’s own framing, meaning that a factor was considered a facilitator if all/most respondents and schools perceive it to be enabling for change commitment or change efficacy. All meaning units were further sorted in Excel to determine which problems or potentials were most prominent at the schools: Similar meaning units that were mentioned most frequently were considered most prominent (see Additional file 3). The Excel file further allowed us to systematically examine differences between schools with and without experiences in implementing SFSH. As we found no major differences in results between schools, the results are not reported separately.

The analysis was presented to and discussed with research and practitioner experts. These discussions further helped in structuring the analysis. Further, all study participants were asked to read and approve their quotations in the analytical content. Thus, the study findings have been triangulated [44] using both expert and target group feedback.

## Results

We identified 13 facilitators and barriers to change commitment or change efficacy as shown in Table 3. These factors are thus important for developing organizational readiness to implement SFSH in Danish vocational schools.

**Table 3:** Overview of barriers to and facilitators for either change commitment or change efficacy

	Change commitment	Change efficacy
**Facilitators**	• Health Promotion is a school role and duty	• Clear rules and responsibilities in sanctioning and enforcement
	• Smoke-free norms are a part of the future (or present) work life, which students need to be prepared for	• Developing a joint understanding as a prerequisite for smoke-free school hours implementation
	• Smoke-free school hours as a strategy for achieving fewer educational interruptions	• Developing skills and confidence to deal with student responses to smoke-free school hours
		• Establishing alternatives to smoking communities at school
		• Offering smoking cessation help or other help for students to cope with smoke-free school hours
		• If smoke-free school hours is decided by law
**Barriers**	• Smoke-free school hours violate personal freedom	• Difficult to administer the level of sanctioning and enforcement
	• Students have more important problems than smoking	• Enforcement might negatively influence student-teacher relations

### Change commitment

A primary facilitating factor for change commitment is believing that the school has a role and duty to promote health and contribute to smoking prevention. More specifically, whether or not their smoking prevention responsibility entails establishing SFSH to avoid having new (young) students initiate or increase smoking when they enroll at the respective school:

It [health promotion] has become a school responsibility (…) to be ahead as a smoke-free school (Manager School F).

The reason why we chose to have smoke-free school hours was not to put pressure on those who smoke; it was to not make new smokers. Because we found out that many new students who started at our school began to smoke while at the school (Teacher School A).

At all the schools, the motivation to establish SFSH further reflect trends in the surrounding society and the different main subject areas, as these represent different types of workplaces and cultures. At schools B and C (representing Care, health and pedagogy), SFSH is seen as a meaningful initiative, as many of the workplaces they are educating the students to join already have or are in the process of establishing smoke-free work hours (e.g., hospitals, municipalities and child care services):

I have to say, what really made an impact was that our municipality established a smoking ban during work hours for all employees (Manager School C).

This is also acknowledged as a motivating factor at schools D and F, which represent the construction sector (technology, construction and transportation) and the business sector (administration, commerce and business), respectively:

You can’t smoke if you are employed to work on [state construction sites, like] the metro… (…) so I suppose it is a good thing (…) if you don’t smoke (Manager School D).

However, it was articulated at school E (Food, agriculture and hospitality) that the hospitality sector (e.g., restaurants) is not becoming smoke-free:

I think as a vocational school (…) you need the profession (…) to support it, like they have smoke-free-work-hours (…) at the hospitals (…), but in restaurants it is okay that you go to smoke out in the back (Manager School E).

Thus, the motivation to establish SFSH is influenced by the smoking practices and cultures at apprenticeships and future workplaces.

Additionally, a perceived benefit of and strategy for establishing SFSH is that school class pedagogy will no longer be influenced by students’ constant need for cigarettes or “smoking breaks”, which means fewer disruptions during teaching:

It is an advantage on the teacher side to be able to keep the students in class and not have them asking for smoking breaks all the time (Teacher School C).

A primary barrier to change commitment is believing that SFSH violate personal freedom. The argument asserts that the school has a responsibility to enlighten or inform their students about health but should not take away individual choices by introducing SFSH:

I think we have a great responsibility [for smoking prevention] on the informational level. (…) But I have to say, personally, I have never actually been a fan of a total smoking ban. I just haven’t. I think it is an interference with personal freedom. (Manager School C).

At school B, many organizational members believed that SFSH violated personal freedom, which led to great resistance among employees, and the school management withdrew the policy after a 6-month period. The school manager described the situation as a fight about values:

I dare to claim that our employee group was divided in two camps independent of smoking status: “Do you believe it’s okay that we as school make these kind of rules?” or “are you opposed to making these kind of rules?” (Manager School B).

Another barrier to change commitment is believing that smoking is not a sufficiently important issue to address because many vocational students have other challenges, such as mental health problems or weed addiction:

The smallest concern for them is if they smoke a cigarette (Teacher School A).

### Change efficacy

Having clear rules and responsibilities regarding sanctioning and enforcement is seen as a prerequisite for implementing SFSH:

So, we had to clear it with our staff on “how do we do this?”: Rules, framings and clarity (…); when you’re caught smoking during school hours, you’re sent home (Manager school A).

At both schools that have implemented SFSH, the responsibilities for enforcement and sanctioning are formulated as part of the schools’ rules of conduct. This means that the organizational members are obligated, as part of their professional tasks, to either send students home from school or give them an oral or written warning if the students are caught smoking during school hours. In contrast, no clear responsibilities or sanctioning procedures were in place at school B, as the school management believed it conflicted with the school’s organizational values:

If we were to go and enforce these rules [SFSH] and there is a negative consequence to it, our organization would really struggle with that. But it was what they [the students] needed (Manager B).

Consequently, formulating clear rules for sanctioning and enforcement facilitates change efficacy, and this needs to be negotiated so that organizational members understand their responsibilities.

Another important facilitator for change efficacy is that schools must develop a joint understanding about SFSH. The argument is that implementation is a “team sport”, and if there is no joint understanding, implementation is impossible:

It is apparent – I can’t implement anything at this school if I don’t have my employees with me (Manager School D).

I think it was that we didn’t have a joint understanding about it; like it wasn’t something where we all held each other’s hands saying, “this is a good idea” (…); there was a REALLY big controversy about it (Teacher School B).

Additionally, developing skills and confidence to deal with student responses to SFSH facilitates change efficacy:

You have to equip the teachers to acknowledge that it is damn hard and sometimes conflictive [SFSH] (…) and make sure that they feel confident and have the right skills and know how to react (Manager School A).

Respondents articulated that smoking has a social function at vocational schools and replacing “smoking communities” with other activities is negotiated as a facilitating strategy to improve the implementation of SFSH:

I think it [smoking] has a social function and attraction (...); therefore, I like the idea of (…) creating other social magnets. It could be (…) establishing physical structures, which calls on being social together without smoking (Manager School F).

Offering smoking cessation help is seen as a necessary supporting action if/when implementing SFSH, as many vocational students are addicted to cigarettes:

We presented it as part  of our introduction, where we say, “this is a smoke-free school, but it is possible to attend smoking cessation courses” (Manager School C).

I think it should be a law [if implementing SFSH] that you’d have to offer smoking cessation courses (Teacher School E).

Legislation on SFSH is perceived as facilitating^1^ change efficacy in two ways. First, it will eliminate a potential negative competitive factor for schools’ ability to attract new students, and second, a law mandating SFSH will help with communication in enforcement situations, allowing managers and staff to place responsibility on the state rather than the school:

If there really are students calculating, “okay, in that school we can smoke, and in that school we can’t” (Manager School C).

I need to be able to say, “it is forbidden [by law] to smoke here” (Manager School E).

A primary barrier to change efficacy is how to administer sanctioning and enforcement. Even though SFSH is practiced at schools A and C, it is well-known that some students still smoke during school hours; therefore, managing enforcement is a daily challenge:

Sometimes they come in (…) and we can smell what they have been up to. IP2: But we must catch them in the act. IP1: (…) so how do we administer the level of our interference? (…) It is something we discuss among the staff (Teachers School C).

Likewise, defining well-functioning enforcement and sanctioning procedures at schools without experience implementing SFSH is recognized as a barrier.

Another barrier to change efficacy is that some believe or have experienced that enforcement negatively influences student-teacher relations. In various cases, a police officer narrative was expressed, and it was argued that enforcement might damage important student-teacher relations:

In this school, we don’t go around keeping eye on the students (…) I would rather talk with the students about if its good weather or if they had a good class (…) The staff (…) have the same dilemma (…) we want to keep good relations with our students (Manager D).

## Discussion

Our study aim was to identify and understand the barriers to and facilitators for developing organizational readiness to implement SFSH. We did so by investigating the facilitators for and barriers to change commitment and change efficacy based on the theory of organizational readiness for change [16]. We identified a total of 13 factors: nine facilitators and four barriers. In the following, we discuss the implications of our use of the organizational readiness for change theory as a guiding theory and discuss the study findings within the context of the extant literature as well as identify our unique contributions.

The theory of organizational readiness for change was relevant and applicable for generating new knowledge about what facilitates or hinders change commitment and change efficacy. It provided a directly operationalizable framework to analyze verbal statements as social-cognitive properties, such as how motivation, willingness, and ability are influenced by organizational context. As such, we found it straightforward to contextualize the concepts of change commitment and change efficacy into a qualitative interview guide. It is our assessment that the basic structure of our operationalization is applicable for use by other researchers. However, it is crucial that the interview guide must be adapted to its context. In this study, we did not identify any factors that could not be explained by the theory. In particular, the use of this theory in relation to school B gave insight into the importance of having enough organizational readiness. Our analysis showed that school B had not developed sufficient change commitment to support implementation of SFSH, as many organizational members believed SFSH violated personal freedom. The school had also not developed sufficient change efficacy, as it did not develop a shared understanding about why the school was implementing SFSH or formulated clear responsibilities for enforcement and sanctioning. The theoretical lens thus offered a plausible explanation for the unsuccessful implementation of SFSH as an expression of insufficient organizational readiness for change. As such, our analysis highlights that organizational readiness is important for schools when implementing SFSH, also identifying specific barriers and facilitators to be addressed.

We identified two antagonistic discourses as prominent factors for change commitment: (1) believing that health promotion and smoking prevention is a school role and duty, which legitimizes SFSH, versus (2) believing that SFSH violate personal freedom. The first discourse corresponds with previous research showing that staff enforcement depends on whether staff members believe that STP enforcement is their role and duty [9], whereas the second discourse has been identified as a general barrier to efforts to control environmental tobacco smoke [45] and as a specific hindering belief for implementing SFSH in the Netherlands [27]. Moreover, the discourses can be said to echo a broader health-political debate concerning which means and models are most appropriate for health promotion and tobacco control [46, 47].

As with other research on STPs [23, 25, 27], we identified staff and managerial perceptions that students’ smoking behavior is a less important issue to address than other health behaviors, such as students’ well-being, as a barrier to change commitment. Moreover, in line with other research [17, 19, 40], our study shows that enforcement is seen as a practice that might negatively affect teacher-student relations. Our study suggests that schools’ efforts to educate or equip staff to deal with student responses to enforcement facilitate change efficacy. This corresponds with the realist review, which contends that staff enforcement depends on whether staff members have confidence to deal with students’ negative responses [9]. Additionally, we found that establishing smoking cessation assistance or other help for nicotine-dependent students when implementing SFSH is seen as a necessary action, and this has also been suggested in similar research [27].

Our study shows that, even though SFSH is practiced at two schools, some students smoke during school hours, and it is thus a daily struggle to manage the level of sanctioning and enforcement. Likewise, international research shows [7, 48] that even at schools with well-implemented STPs, not all students comply with the smoke-free restrictions. We will add that the daily challenge for staff to minimize smoking during school hours might help to transition SFSH as part of routine practice, by ensuring that enforcement practices are being maintained and developed. As such, even though it is a barrier to change efficacy to manage the level of enforcement, it might be a necessary part of the implementation work.

We found that developing a shared understanding among staff and managers about why the school has chosen to implement the policy is seen as a prerequisite for implementing SFSH. Ensuring staff buy-in by developing a shared vision and understanding is known to be a key factor for successful organizational implementation [37], which we identified as a facilitating practice specifically in relation to SFSH. Samdal and Rowling [20] highlight that writing a policy into school documents is a way to ensure institutional anchoring, and it can be described as an “add-in” strategy [49] which emphasizes integrating the new policy into existing school practices and rules of conduct. Similarly, we determined that having clear responsibilities and procedures for sanctioning and enforcement integrated into schools’ rules of conduct facilitates change efficacy.

Among new insights with regard to change readiness at vocational schools, we found that SFSH is seen as meaningful if the policy aligns with the (smoking) culture, norms, and practices at the workplaces for which the vocational schools are educating students. Subsequently, change commitment to SFSH is stronger if the apprenticeship-workplace has already established smoke-free work hours and weak if smoking is normal or accepted during work hours.

Moreover, our study demonstrates that smoking is seen as a social problem at Danish vocational schools and that students’ asking for “smoking breaks” takes up time and energy in the classroom. To our knowledge, our study is the first to identify SFSH as a strategy for achieving fewer educational interruptions and the first to suggest that establishing alternatives to smoking communities might ease the implementation of SFSH. Intervention research at Danish vocational schools has shown effectiveness to reduce occasional smoking, when new school break activities are introduced in combination with decreasing the number of (smoking) school breaks [50, 51]. This validates efforts to structurally change social patterns as an appropriate strategy when/if implementing SFSH.

The Danish SFSH law — which will be ratified in August 2021 — consolidates the decision from the school to the state level. However, the challenge of implementation remains unchanged: The policy must be locally enacted by school organizational members and experienced by students. Evidence shows that change-related efforts based on “want to” motives (individuals value the change) promote implementation more effectively than decisions based on “we have to” motives (individuals have little choice) [38]. Hence, despite the law, it might be beneficial to develop organizational readiness by stimulating change commitment. Our study was conducted before the SFSH policy was decided by law but showed that the law can facilitate change efficacy in two ways: It means that all vocational schools have the same conditions in terms of attracting new students, and it can be used communicatively in enforcement situations, whereby staff and managers can justify SFSH by referring to the law. To our knowledge, this is the first study to explain why and how a SFSH law can ease implementation.

### Strengths and limitations

Within the fixed study design (6 schools), we did not apply a data saturation method, which is a limitation. However, we used both expert and target group feedback as a triangulation-of-results method, which suggests that our findings are valid and transferable to other Danish vocational schools. In addition, we found similar discourses at schools with and without experiences implementing SFSH, which implies that data saturation has been reached. Moreover, many barriers and facilitating factors have been recognized in international research on STPs, suggesting that our results are transferable to similar countries.

We were not able to recruit enough participants for some of the focus groups, which resulted in reduced dialog and discussions, which is a methodological limitation.

### Implications for research and practice

This study identified barriers and facilitators that are important for developing organizational readiness to implement SFSH. These learnings have been integrated into the Smoke-Free Vocational Schools intervention project, which is taking place in 11 Danish vocational schools from 2018 to 2022. Details on the development of this study has been described elsewhere [52]. Briefly, integration of results was secured at a workshop where research and practice partners together developed the core elements of the intervention, e.g., it was decided that the intervention includes a 6-month preparation phase, where intervention activities, (e.g., workshops, facilitation in communication strategies), are delivered at the schools to stimulate organizational readiness to implement SFSH. For example, the preparation phase includes an explicit focus on stimulating a joint vision and understanding of why the school is implementing SFSH and having clear rules and responsibilities for sanctioning and enforcement as well as structurally establishing alternatives to smoking communities at school [52]. To this date, no other interventions has been developed to increase organizational readiness for change [18]. As such, this study has informed the development of an intervention for implementing SFSH and thus aligns with the implementation science aim of translating research into practice [53]. There are still several unanswered questions and possible challenges in relation to implementing SFSH. We have determined that clear rules for sanctioning and enforcement are important but not which rules are most beneficial nor how schools can incorporate them. Likewise, we found that it is appropriate to offer smoking cessation help but not which form of help is most appropriate or how schools can integrate the assistance into their routine practices. Further research should thus focus on how to strengthen the identified facilitators and counter the barriers.

Other scholars have provided general instruments to measure organizational readiness for change [18], but our study suggests that there are multiple, context-specific facilitators and barriers, which may not be the same at every site, that needs to be addressed when building readiness for SFSH. Thus, it is important to adapt general instruments to the specific context and/or the specific school type (e.g., VET, university, primary school), also allowing an openness to capture the possible site-specific facilitators and barriers. As such, our study builds on other research related to organizational readiness for change, where social scientists have found it relevant to develop new or adopt existing scales for their specific research aims [18]. However, many of the scales used have unknown or insufficient psychometric properties [18]; thus, further research should apply validated instruments to measure organizational readiness. Besides, only few existing studies have assessed associations between organizational readiness and outcomes, e.g., successful implementation [18]. Future research should investigate the relation between SFSH and successful implementation using a validated instrument, which also allows for the measuring of context-specific facilitators and barriers. Such an instrument could shed light on which facilitators and barriers are most frequent and further allow researchers to investigate if there is a threshold of organizational readiness that should be meet prior to initiating implementation of SFSH [18], e.g., do all organizational members need to be ready at the same time, or is readiness more important among specific groups or individuals?

Data from this study was collected in 2018 and in late 2020, a Danish law was passed ratifying SFSH at primary and secondary schools among students (not staff, thus a less comprehensive policy than the one investigated in this paper). Despite a law now has been passed that also applies to vocational schools, this does not eliminate the need for schools to make sure that organizational readiness to implement SFSH is present to secure a successful implementation of SFSH. It is obvious that many of the identified facilitators and barriers may still be present at vocational schools and similar settings and need to be addressed for a law to be implemented and enforced successfully.

## Conclusion

This study demonstrates the importance of developing organizational readiness when implementing SFSH. Among the most prominent facilitators for developing readiness are believing that SFSH is part of a school’s health promotion role and duty and that schools have clear responsibilities with regard to sanctioning and enforcement. The most important barriers include believing that SFSH violate personal freedom and believing that enforcement is not possible and not worthwhile.

## Supplementary Information

**Additional file 1.** Reporting Standards

**Additional file 2.** Operationalization of the Organizational Readiness for Change theory to interview guides

**Additional file 3.** Coding three

## Data Availability

The datasets generated and analyzed during the current study are not publicly available due to the identifiable nature of qualitative data.

## Abbreviations

SFSH: Smoke-free school hours
STP: School tobacco policy
VET: Vocational education and training

## Footnotes

1 In Denmark, smoke-free school hours will be ratified by law in 2021; therefore, it is categorized as a facilitating factor.
