# Supplementary material for: Are Danish vocational schools ready to implement “smoke-free school hours”? A qualitative study informed by the theory of organizational readiness for change
Source: Implement Sci Commun. 2021 Apr 9;2:40. doi: 10.1186/s43058-021-00140-x (PMC8033695; doi:10.1186/s43058-021-00140-x)
Supplement: Supplementary file 1 — Additional file 1. Reporting Standards [file 43058_2021_140_MOESM1_ESM.docx]

**Additional file 1: Reporting Standards**

| Section | COREQ | SRQR | How the manuscript adheres to the reporting standards |
| --- | --- | --- | --- |
| **Title** | N/A | S1 Concise description of the nature and topic of the study Identifying  the study as qualitative or indicating the approach (e.g., ethnography,  grounded theory) or data collection methods (e.g., interview, focus  group) is recommended | We describe the nature and topic of the study. |
| **Abstract** | N/A | S2 Summary of key elements of the study using the abstract format of  the intended publication; typically includes background, purpose,  methods, results, and conclusions | We summarize key elements of the study using the abstract format. |
| **Background** | N/A | S3 Description and significance of the problem/phenomenon studied;  review of relevant theory and empirical work; problem statement  S4 Purpose of the study and specific objectives or questions | We describe both our problem studied and a review of relevant literature.  We describe the purpose of the study and specific research question. |
| **Background** Context | N/A | S7 Setting/site and salient contextual factors; rationale | We describe the context (vocational schools) in the Background section and in the Methods section. SRQR proposes it is described under Methods. |
| **Background**  Qualitative approach and research paradigm | What methodological orientation was stated to underpin the study? e.g. grounded theory,  discourse analysis, ethnography, phenomenology, content analysis | S5 Qualitative approach (e.g., ethnography, grounded theory, case study,  phenomenology, narrative research) and guiding theory if appropriate;  identifying the research paradigm (e.g., postpositivist, constructivist/  interpretivist) is also recommended; rationale | We include a ‘Theoretical Framework’ section where we describe that our methodological orientation and guiding theory is the social-cognitive A theory of organizational readiness for change.  In our manuscript, this is part of the Background section as it used to explain our research question. SRQR and COREQ proposes it is described under Methods. |
| **Methods**  Researcher characteristics and reflexivity | Characteristics  1. Interviewer/facilitator Which author/s conducted the interview or focus group?  2. Credentials What were the researcher’s credentials? E.g. PhD, MD  3. Occupation What was their occupation at the time of the study?  4. Gender Was the researcher male or female?  5. Experience and training What experience or training did the researcher have?  Relationship with participants  6. Relationship established Was a relationship established prior to study commencement?  7. Participant knowledge of the  interviewer  What did the participants know about the researcher? e.g. personal goals, reasons for doing the  research  8. Interviewer characteristics What characteristics were reported about the interviewer/facilitator? e.g. Bias, assumptions,  reasons and interests in the research topic | S6 Researchers’ characteristics that may influence the research, including  personal attributes, qualifications/experience, relationship with  participants, assumptions, and/or presuppositions; potential or actual  interaction between researchers’ characteristics and the research  questions, approach, methods, results, and/or transferability | We describe who conducted the interviews and focus groups, including credentials, occupation, gender and experience/training in qualitative research.  We describe how the relationship with participations was established and that the researchers had no relationship with the participants before study commencement.  We do NOT describe other interviewer characteristics e.g. bias, assumptions, reasons and interests in the research topic. Not relevant. |
| **Methods**  Sampling and recruitment | Participant selection  10. Sampling How were participants selected? e.g. purposive, convenience, consecutive, snowball  11. Method of approach How were participants approached? e.g. face-to-face, telephone, mail, email  12. Sample size How many participants were in the study?  13. Non-participation How many people refused to participate or dropped out? Reasons? | S8 How and why research participants, documents, or events were  selected; criteria for deciding when no further sampling was necessary  (e.g., sampling saturation); rationaleb | We describe that we use a purposive sampling strategy with preselected criteria.  We describe how participants were recruited (email and telephone).  We describe the sample size and that we included two respondent groups (teachers and managers).  We describe that all invited schools accepted the participation invitation, but not the desired number of teachers where able to participate in study due to busy schedules. The later is reported as a methodological limitation under Strengths and Limitations in the Discussion section. |
| **Methods**  Setting and description of sample | Setting  14. Setting of data collection Where was the data collected? e.g. home, clinic, workplace  15. Presence of non-participants Was anyone else present besides the participants and researchers?  16. Description of sample What are the important characteristics of the sample? e.g. demographic data, date | S12 Number and relevant characteristics of participants, documents, or  events included in the study; level of participation (could be reported  in results) | We describe that data collection takes place in offices where participants felt comfortable.  We describe which researchers collected the data (see Researcher Characteristics).  Table 1 include number of participants and relevant participants characteristics.  We describe which year and months the interviews take place.  We do NOT include participants’ gender and age in Table 1, as we do not believe their demographic to influence the research question. |
| **Methods**  Data collection and data processing | Data collection  17. Interview guide Were questions, prompts, guides provided by the authors? Was it pilot tested?  18. Repeat interviews Were repeat interviews carried out? If yes, how many?  19. Audio/visual recording Did the research use audio or visual recording to collect the data?  20. Field notes Were field notes made during and/or after the interview or focus group?  21. Duration What was the duration of the interviews or focus group?  22. Data saturation Was data saturation discussed?  23. Transcripts returned Were transcripts returned to participants for comment and/or correction? | S10 Types of data collected; details of data collection procedures including  (as appropriate) start and stop dates of data collection and analysis,  iterative process, triangulation of sources/methods, and modification  of procedures in response to evolving study findings; rationale  S11 Description of instruments (e.g., interview guides, questionnaires)  and devices (e.g., audio recorders) used for data collection; if/how the  instrument(s) changed over the course of the study  S13 Methods for processing data prior to and during analysis, including  Transcription. | We describe the data collection methods (semi-structured interviews and focus groups).  We describe interview guides including topics and questions.  We describe the qualitative format: semi-structured interview and focus group including why we chose the format.  We describe that data was audio recorded and the durations of the interviews/focus groups.  We describe, that data was transcribed verbatim.  The transcripts were NOT returned to the participants for comments and/or correction. The interview guide was NOT piloted, nor did we use field notes. Neither was necessary in our study.  We did NOT do repeat interviews to ensure data saturation, which is a limitation – discussed under Strengths and Limitations in the Discussion section. |
| **Methods**  Research ethics and data management | N/A | S9 Ethical issues pertaining to human subjects.  S13 Data entry, data management and security, verification  of data integrity, data coding, and anonymization/deidentification of excerpts | We describe that oral informed consent was obtained prior from all participants prior to the interviews.  We describe our data management procedures including security and that data is pseudo-anonymized.  We describe that our study has been reported to Capital Region of Denmark’ legal centre for personal data handling, according to GDPR, which is appropriate in Denmark. |
| **Methods**  Data analysis | Data analysis  24. Number of data coders How many data coders coded the data?  25. Description of the coding tree Did authors provide a description of the coding tree?  26. Derivation of themes Were themes identified in advance or derived from the data?  27. Software What software, if applicable, was used to manage the data?  28. Participant checking Did participants provide feedback on the findings? | S14 Process by which inferences, themes, etc., were identified and  developed, including the researchers involved in data analysis; usually  references a specific paradigm or approach; rationale b  S15 Techniques to enhance trustworthiness and credibility of data analysis  (e.g., member checking, audit trail, triangulation); rationaleb | We describe the number of data coders (one).  We describe the process by with we identified themes in the data (structuring content analysis). Additional file 2 include the ‘coding tree’, which show all themes identified in the data and their frequency.  We describe that we used a deductive analysis strategy.  We describe the software used (NVIVO and Excel).  We describe that we used expert-group and target-group feedback as a method to ensure the reliability of the results. |
| **Results** | Reporting  29. Quotations presented Were participant quotations presented to illustrate the themes / findings? Was each  quotation identified? e.g. participant number  30. Data and findings consistent Was there consistency between the data presented and the findings?  31. Clarity of major themes Were major themes clearly presented in the findings?  32. Clarity of minor themes Is there a description of diverse cases or discussion of minor themes? | S16 Main findings (e.g., interpretations, inferences, and themes); might  include development of a theory or model, or integration with prior  research or theory  S17 Evidence (e.g., quotes, field notes, text excerpts, photographs) to  substantiate analytic findings | Quotations are presented to substantiate the analytical findings. We believe that the results are clearly presented.  It is possible to identify each quotation by school and respondent group (teacher or manager).  It is NOT possible to identify each participant in the focus groups, which is not considered relevant. |
| **Discussion**  Interpretation of findings | N/A | S18 Short summary of main findings; explanation of how findings  and conclusions connect to, support, elaborate on, or challenge  conclusions of earlier scholarship; discussion of scope of application/  generalizability; identification of unique contribution(s) to scholarship  in a discipline or field | We discuss our main findings within the context of the extant literature and identify our unique contributions.  Further, we offer implications for future research and practice. |
| **Discussion**  Limitations | N/A | S19 Trustworthiness and limitations of findings | We describe both study strengths and limitations. |
| **Other** | N/A | S20 Potential sources of influence or perceived influence on study conduct  and conclusions; how these were managed  S21 Sources of funding and other support; role of funders in data  collection, interpretation, and reporting | We declare no conflicts of interests.  The declare, that the study has been supported by the Danish Health Authority. The Danish Health Authority had no role in data collection, interpretation or reporting of the study findings. |
