# Supplementary material for: Are Danish vocational schools ready to implement “smoke-free school hours”? A qualitative study informed by the theory of organizational readiness for change
Source: Implement Sci Commun. 2021 Apr 9;2:40. doi: 10.1186/s43058-021-00140-x (PMC8033695; doi:10.1186/s43058-021-00140-x)
Supplement: Supplementary file 2 — Additional file 2. Operationalization of the Organizational Readiness for Change theory to interview guides [file 43058_2021_140_MOESM2_ESM.docx]

**Additional File 2: Operationalization of the Organizational Readiness for Change theory to interview guides**

Interview guide – Management (Semi-structured interview)

| Concept | Question – examples |
| --- | --- |
| **Theme: Introduction** | |
| Introduction | Information about study purpose, data confidentiality and that participation is voluntary. |
| Background information | What are your primary management responsibilities at the school?  For how long have you had the position at this school?  Do you have experience in integrating health promotion initiatives into the school practice? |
| **Theme: Current school tobacco policy and smoking practices** | |
| CONTEXT: Current school tobacco policy | Which rules about smoking are in place at your school?  Do the rules include school staff?  What tobacco-related products are included by the rules? |
| CONTEXT: Current smoking practice | What is your experience with student adherence to the school tobacco policy? |
| **Theme: Smoke-free school hours** | |
| Introduction to the concept of smoke-free school hours | Information about the definition of smoke-free school hours |
| CHANGE COMMITMENT: Motivation | Why did your school decide to implement smoke-free school hours? / Why do you consider implementing smoke-free school hours? |
| CHANGE COMMITMENT: Change valence | What did/do you expect to gain by implementing smoke-free school hours? |
| CHANGE COMMITMENT: Types of motives | What of the following statements would you say best characterize the motives behind the decision to implement smoke-free school hours and why?   - We *have to* implement smoke-free school hours - We *ought to* implement smoke-free school hours - We *want to* implement smoke-free school hours |
| CHANGE COMMITMENT: School culture | Do you believe that most staff members share the motivation to implement smoke-free school hours, you’ve just described, why or why not? |
| CHANGE COMMITMENT: Openness to change | How would you describe the climate among the staff members with regards to implementing new health promoting initiatives in general? |
| CHANGE COMMITMENT: Responsibility believes | Do you believe that the school has a responsibility to contribute towards smoking prevention, why or why not?  Do you believe that the school has a responsibility to contribute towards smoking cessation, why or why not? |
| CHANGE EFFICACY: Task demands; What does it take to implement smoke-free school hours | Could you describe step-by-step how you implemented smoke-free school hours, including whom you’ve involved and in which processes? / Could you describe how you would go about implementing smoke-free school hours, including whom you’ve would involve and in which processes? |
| CHANGE EFFICACY: Task demands; Skills | What is needed to implement smoke-free school hours?  Do you believe that most staff members have the skills needed to implement smoke-free school hours, why or why not? |
| CHANGE EFFICACY: Task demands; Challenges | What are the most persistent challenges when implementing smoke-free school hours?  How did/would you deal with the challenges? |
| CHANGE EFFICACY: Time and timing | Do you believe that there was/is enough time available to implement smoke-free school hours, why or why not?  Do you think that the timing was/is right to implement smoke-free school hours, why or why not? |
| CHANGE EFFICACY: Resources | Do you think there was/is enough resources to implement smoke-free school hours successfully, why or why not? |
| CHANGE EFFICACY: External influences | How do you consider the reaction among the outside world e.g. the apprenticeship workplaces, when/if your school decided to implement smoke-free school hours? |

Interview guide – Teachers (Semi-structured focus group)

| Concept | Question – examples |
| --- | --- |
| **Theme: Introduction** | |
| Introduction | Information about study purpose, data confidentiality and that participation is voluntary. |
| Background information | Which classes are you teaching at the school?  For how long have you had the position at the school?  Do you have experience in integrating smoking prevention in your teaching? |
| **Theme: Current school tobacco policy and smoking practices** | |
| CONEXT: Current school tobacco policy | Which rules about smoking are in place at your school? |
| CONTEXT: Current smoking practice | What is your experience with student adherence to the school tobacco policy?  How would you describe the smoking practice at the school?  Do you think the current smoking practice at the school is a problem, why or why not? |
| **Theme: Smoke-free school hours** | |
| Introduction to the concept of smoke-free school hours | Information about the definition of smoke-free school hours |
| CHANGE COMMITMENT: Motivation | What do you think about smoke-free school hours in relation to your school? |
| CHANGE COMMITMENT: School culture | Do you believe that most staff members share the believes about smoke-free school hours, you’ve described, why or why not? |
| CHANGE COMMITMENT: Responsibility believes | Do you believe that the school has a responsibility to contribute towards smoking prevention, why or why not?  Do you believe that the school has a responsibility to contribute towards smoking cessation, why or why not? |
| CHANGE COMMITMENT: Change valence | The teachers were asked to fill out the below table and discuss their different point of views.   \| ***Advantages of smoke-free school hours*** \| ***Disadvantages of smoke-free school hours*** \| \| --- \| --- \| \| *For students* \| *For students* \| \| *For teachers* \| *For teachers* \| |
| CHANGE EFFICACY: Task demands; Skills | Do you believe that most staff members have the skills needed to implement smoke-free school hours, why or why not? |
| CHANGE EFFICACY: Task demands; Challenges | What are the most persistent challenges when implementing smoke-free school hours?  How did/would you deal with the challenges? |
| CHANGE EFFICACY: Time and timing | Do you believe that there was/is enough time available to implement smoke-free school hours, why or why not?  Do you think that the timing was/is right to implement smoke-free school hours, why or why not? |
| CHANGE EFFICACY: Resources | Do you think there was/is enough resources to implement smoke-free school hours successfully, why or why not? |
| CHANGE EFFICACY: External influences | How do you consider the reaction among the outside world e.g. the apprenticeship workplaces, when/if your school decided to implement smoke-free school hours? |
