# Supplementary material for: Are Danish vocational schools ready to implement “smoke-free school hours”? A qualitative study informed by the theory of organizational readiness for change
Source: Implement Sci Commun. 2021 Apr 9;2:40. doi: 10.1186/s43058-021-00140-x (PMC8033695; doi:10.1186/s43058-021-00140-x)
Supplement: Supplementary file 3 — Additional file 3. Coding three [file 43058_2021_140_MOESM3_ESM.docx]

| **Meaning units** | **Number of meaning units** | **Number of meaning units by respondent group** | **Meaning units by schools** | **Analysis: B or F** | **Analysis: CC or CE** |
| --- | --- | --- | --- | --- | --- |
| Health promotion and smoking prevention is a school role and duty | 50 | Teacher: 27 Manager: 23 | All | F | CC |
| Smoke-free-school-hours is violating personal freedom | 41 | Teacher: 26 Manager: 15 | All | B | CC |
| Clear rules and responsibilities in enforcement and sanctioning | 31 | Teacher: 15 Manager: 17 | A, B, C, F | F | CE |
| Developing a joint understanding is a prerequisite for smoke-free-school-hours implementation | 30 | Teacher: 14 Manager: 16 | All | F | CE |
| Developing skills and confidence to deal with student responses to smoke-free-school-hours | 25 | Teacher: 16 Manager: 9 | A, C, E | F | CE |
| Smoke-free norms are a part of the future (or present) work life, which students need to be prepared for | 24 | Teacher: 12 Manager: 12 | All | F | CC |
| If smoke-free-school-hours is decided by law | 19 | Teacher: 6 Manager: 13 | B, C, D, E, F | F | CC |
| Difficult to administer the level of enforcement and sanctioning | 18 | Teacher: 14 Manager: 4 | All | B | CE |
| Students have more important problems than smoking | 17 | Teacher: 11 Manager: 6 | A, C, D, E, F | B | CC |
| Establishing alternatives to smoking communities at school | 16 | Teacher: 7 Manager: 9 | A, D, E, F | F | CE |
| Smoke-free-school-hours as a strategy to less educational interruptions in the classroom | 16 | Teacher: 13 Manager: 3 | B, C, D, E | F | CC |
| Enforcement might negatively influence teacher-student relations | 16 | Teacher: 10 Manager: 6 | All | B | CE |
| Offering smoking cessation help or other help to students to cope with smoke-free-school-hours | 14 | Teacher: 6 Manager: 8 | A, B, C, E, F | F | CE |
| The difference between formal smoke-free-school-hours agreement and making smoke-free-school-hours a part of routine practice | 11 | Teacher: 4 Manager: 7 | All | B | CE |
| Smoke-free-school-hours might cause student drop-out | 11 | Teacher: 6 Manager: 5 | A, C, E, F | B | CC |
| Not enough time nor resources – smoke-free-school-hours is not a good priority | 10 | Teacher: 4 Manager: 6 | D, E, F | B | CE |
| Dealing with smoke-free-school-hours enforcement – staff doesn’t personally know all students | 8 | Teacher: 4 Manager: 4 | A, C, F | B | CE |
| Creating a better physical environment without smoke nuisances | 4 | Manager: 4 | C, D, E | F | CC |
| Smoke-free-school-hours is a better alternative than banning smoking from school grounds | 4 | Teacher: 1 Manager: 3 | C, D, F | F | CC |
| Smoke-free-school-hours is a part of routine practice | 4 | Teacher: 4 | A, C | F | CE |
| Smoke-free-school-hours can create a school health promotion image | 4 | Teacher: 2 Manager: 2 | C, D, F | F | CC |
| External support in smoke-free-school-hours implementation | 3 | Manager: 3 | B, C, D | F | CC |

Additional file 2: Coding three
